# Cleavage-stage embryo segmentation using SAM-based dual branch pipeline: development and evaluation with the CleavageEmbryo dataset

**DOI:** 10.1093/bioinformatics/btae617

**Published:** 2024-10-18

**Authors:** Chensheng Zhang, Xintong Shi, Xinyue Yin, Jiayi Sun, Jianhui Zhao, Yi Zhang

**Affiliations:** School of Computer Science, Wuhan University, Wuhan, Hubei 430072, China; First School of Clinical Medicine, Wuhan University, Wuhan, Hubei 430060, China; First School of Clinical Medicine, Wuhan University, Wuhan, Hubei 430060, China; First School of Clinical Medicine, Wuhan University, Wuhan, Hubei 430060, China; School of Computer Science, Wuhan University, Wuhan, Hubei 430072, China; Reproductive Medical Center, Renmin Hospital of Wuhan University, Wuhan, Hubei 430060, China

## Abstract

**Motivation:**

Embryo selection is one of the critical factors in determining the success of pregnancy in in vitro fertilization procedures. Using artificial intelligence to aid in embryo selection could effectively address the current time-consuming, expensive, subjectively influenced process of embryo assessment by trained embryologists. However, current deep learning-based methods often focus on blastocyst segmentation, grading, or predicting cell development via time-lapse videos, often overlooking morphokinetic parameters or lacking interpretability. Given the significance of both morphokinetic and morphological evaluation in predicting the implantation potential of cleavage-stage embryos, as emphasized by previous research, there is a necessity for an automated method to segment cleavage-stage embryos to improve this process.

**Results:**

In this article, we introduce the SAM-based dual branch segmentation pipeline for automated segmentation of blastomeres in cleavage-stage embryos. Leveraging the powerful segmentation capability of SAM, the instance branch conducts instance segmentation of blastomeres, while the semantic branch performs semantic segmentation of fragments. Due to the lack of publicly available datasets, we construct the CleavageEmbryo dataset, the first dataset of human cleavage-stage embryos with pixel-level annotations containing fragment information. We train and test a series of state-of-the-art segmentation algorithms on CleavageEmbryo. Our experiments demonstrate that our method outperforms existing algorithms in terms of objective metrics (mAP 0.874 on blastomeres, Dice 0.695 on fragments) and visual quality, enabling more accurate segmentation of cleavage-stage embryos.

**Availability and implementation:**

The code and sample data in this study can be found at: https://github.com/12austincc/Cleavage-StageEmbryoSegmentation.

## 1 Introduction

In vitro fertilization (IVF) is a highly effective assisted reproductive technology used to treat infertility. Identifying embryos with high implantation potential is crucial for ensuring a successful pregnancy. After fertilization, zygotes typically undergo cleavage, compaction, and blastocyst formation before implantation. Therefore, assessing the quality of cleavage-stage embryos is a vital step in embryo development, as it helps predict their implantation potential. This evaluation is usually performed at various time points and involves assessing several morphological parameters (Balaban *et al.* 2011). Currently, trained embryologists manually review images or time-lapse videos of developing embryos. However, this approach is both time-consuming and labor-intensive, often leading to inconsistent inter- and intra-observer agreement and introducing significant uncertainties (Storr *et al.* 2017).

In recent years, deep learning has shown great potential in medical image analysis, leading to the exploration of various AI-based methods to improve embryo selection. Some approaches focus on the blastocyst stage, segmenting blastocysts to accurately identify trophectoderm (TE), zona pellucida (ZP), inner cell mass (ICM), and blastocoel (BL) (Saeedi *et al.* 2017, Arsalan *et al.* 2022). These methods aim to correlate these parameters with implantation potential or to grade embryos (Bormann *et al.* 2020, Thirumalaraju *et al.* 2021). However, they often overlook the morphokinetic and morphological information from the earlier cleavage stage. Other methods (Tran *et al.* 2019, Kragh *et al.* 2022) directly use time-lapse videos to predict embryo outcomes but may lack clear decision logic and interpretability. Alternatively, some methods such as Sharma *et al.* (2023) focus on morphokinetic parameters by analyzing time-lapse videos to determine cell cleavage timings, yet they do not account for morphological details such as blastomere size and fragmentation during cleavage-stage development. In general, these methods tend to neglect the morphological details of cleavage-stage embryos, despite findings by Jiang *et al.* (2022) emphasizing the importance of such evaluations for predicting implantation potential.

In this study, we hope to automatically segment cleavage-stage embryos to obtain detailed information such as the number of blastomeres and the percentage of fragments to assist in assessing their developmental potential. As no existing studies have specifically addressed cleavage-stage embryo segmentation, we draw on cell instance segmentation, which shares great similarities, as a potential source of transferable methods. The majority of mainstream approaches fall into two categories, one is the region proposal-based methods, which generate candidate bounding boxes through object detection and classify object pixels based on these boxes, such as the classical Mask R-CNN instance segmentation framework (He *et al.* 2017) and, for instance, Prangemeier *et al.* (2020) proposed Cell-DETR which applies the object detection framework DETR to cell segmentation; another is segmentation-based approaches which use a combination of pixel-level models such as U-Net (Ronneberger *et al.* 2015) and post-processing techniques to achieve instance segmentation. For example, Lu *et al.* (2015) utilized a shape prior constraint to refine the cytoplasmic boundaries to segment overlapping clusters of cells, and Song *et al.* (2017) used a dynamic multi-template deformation model for boundary refinement and inference. While these approaches have proven effective in general cell instance segmentation, the particular characteristics of overlapping blastomeres and fragment coverage in embryo images may limit their effectiveness. Our work aims to fill this gap. Due to the issue of high overlap (over 50%) caused by cell clustering in embryos, segmentation-based methods have difficulty achieving our purpose. Inspired by the zero-shot instance segmentation approach of SAM, we design a region proposal-based method in collaboration with SAM.

Recently, the prompt-based Segment Anything Model (SAM) (Kirillov *et al.* 2023), trained over 1 billion masks on 11 million images, excels in prompt-based segmentation with strong zero-shot generalization. Studies (Wu *et al.* 2025, Ma *et al.* 2024) have applied SAM to segment tumors, organs, and other anatomical structures, proving its robustness in medical image segmentation. Pandey *et al.* (2023) combined Yolov8 with SAM for precise segmentation, demonstrating the promise of the Yolov8+SAM model for medical image segmentation. However, the experimental results (He *et al.* 2023, Mazurowski *et al.* 2023) indicate that SAM, without fine-tuning, may not be as accurate as specialized algorithms on some datasets. Furthermore, SAM’s accuracy heavily relies on prompts, with bounding box prompts yielding significantly better results than point prompts.

Based on existing research, we design our pipeline which uses a detection model to get bounding box prompts of the instances for guiding the fine-tuned SAM to produce segmentation results. It can work well on the instance segmentation of blastomeres. However, while SAM can achieve satisfactory results with accurate bounding box prompts, the limitations of the detection model in accurately identifying fragments may lead to suboptimal outcomes. Importantly, precise identification of individual fragments is not necessary since our focus lies in assessing the proportion and distribution of fragments, which are vital for evaluating embryo quality. Consequently, semantic segmentation is more suitable for fragments, as it enables us to capture their overall coverage and distribution without the need to treat each fragment as a separate instance. Therefore, we specifically design a fragment branch utilizing semantic segmentation, improving segmentation performance by focusing on fragment regions. It is notable that given the importance of accurately determining the number, shape, and size of each blastomere, instance segmentation remains essential for blastomeres.

The powerful capabilities of deep learning models require excellent algorithms and extensive datasets. To the best of our knowledge, there are currently only three publicly accessible datasets concerning human embryos. One dataset provides time-lapse videos of developing embryos (Gomez *et al.* 2022) and two datasets consist of static blastocyst images with annotations (Saeedi *et al.* 2017, Kromp *et al.* 2023). While the time-lapse video dataset has made a valuable contribution to the field of deep learning-based embryo development assessment, its lack of annotations limits model training. Although the two blastocyst image datasets include annotations, they are somewhat inadequate in comparison to the pixel-level annotations and they do not include cleavage-stage embryos. Therefore, we construct CleavageEmbryo, a cleavage-stage embryo dataset with pixel-level annotations.

In this article, we introduce the SAM-based Dual Branch Segmentation Pipeline for automated segmentation of cleavage-stage embryos, with a semantic branch to segment fragments and an instance branch to detect and segment blastomeres, resulting in the automatically fused segmentation of cleavage-stage embryos. In addition, we construct the CleavageEmbryo dataset, which to the best of our knowledge is the first human embryo dataset with pixel-level annotations that take fragment information into account. We train and benchmark both classic and advanced segmentation algorithms along with our method on the CleavageEmbryo dataset. Experimental results show that our method outperforms other algorithms in terms of objective metrics (mAP 0.874 on blastomeres, Dice 0.695 on fragments) and subjective visual assessments.

## 2 Materials and Methods

### 2.1 Overall architecture

**Figure 1.**
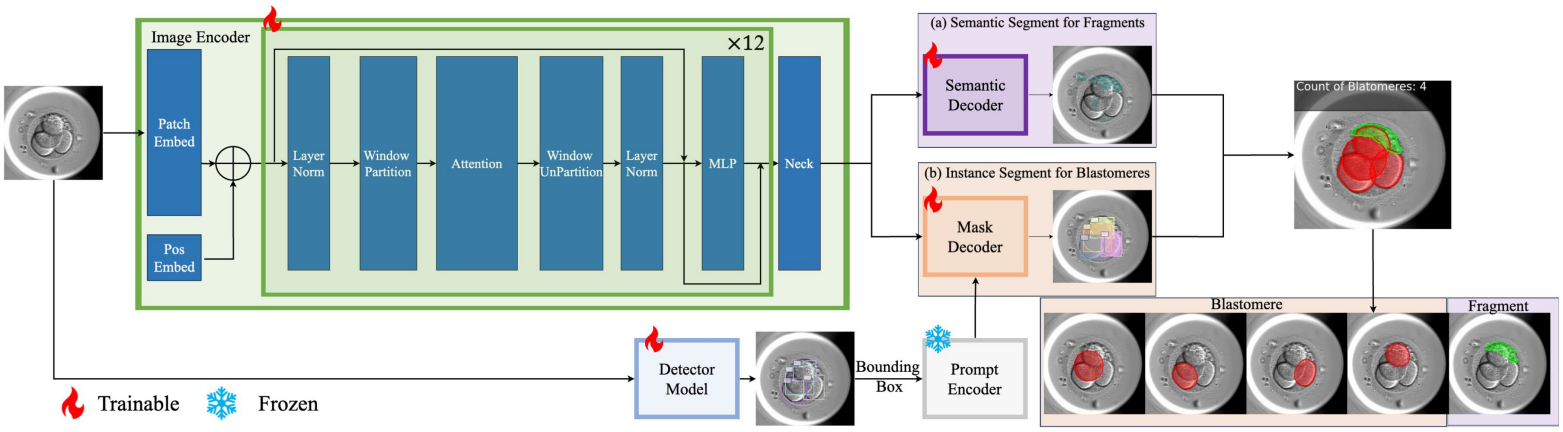
Overall architecture. The pipeline integrates semantic and instance segmentation for cleavage-stage embryos. It begins with the Image Encoder extracting high-dimensional Image Embeddings. These are processed in parallel by the (a) Semantic Branch to segment fragments (shown with green masks and green boundary) and the (b) Instance Branch to detect and segment individual blastomeres (each instance is shown with red mask and red boundary). The results are fused to produce a comprehensive segmentation mask.

Our overall architecture is illustrated in Fig. 1. Considering the unique characteristics of cleavage-stage embryo cells, our pipeline integrates semantic segmentation and instance segmentation to accurately segment both blastomeres and fragments in cleavage-stage embryos.

The process begins with the Image Encoder, which extracts high-dimensional Image Embeddings from the input image. These embeddings are then utilized in two parallel branches: the Semantic Branch and the Instance Branch. In the Semantic Branch, the Image Embeddings are fed into the Semantic Decoder to produce a segmentation mask for fragments within the image. Concurrently, the Instance Branch uses the Detector Model to identify individual blastomeres and generate bounding boxes. Prompt Decoder then processes these bounding boxes to generate Prompt Embeddings. These embeddings, combined with Image Embeddings, are used by the Mask Decoder to generate instance segmentation masks for each detected blastomere.

Finally, the semantic segmentation mask and the instance segmentation masks are fused to create a comprehensive segmentation result that accurately represents both blastomeres and fragments.

The overall loss function for the pipeline is computed as follows:

(1) $$L=L_{\mathrm{Sem}}+L_{\mathrm{Ins}}$$

where $L_{\mathrm{Sem}}$ refers to the loss of Semantic Branch, which is computed as Equation (2) and $L_{\mathrm{Ins}}$ refers to the loss of Instance Branch, which is computed as Equation (3).

### 2.2 Semantic segment for fragments

**Figure 2.**
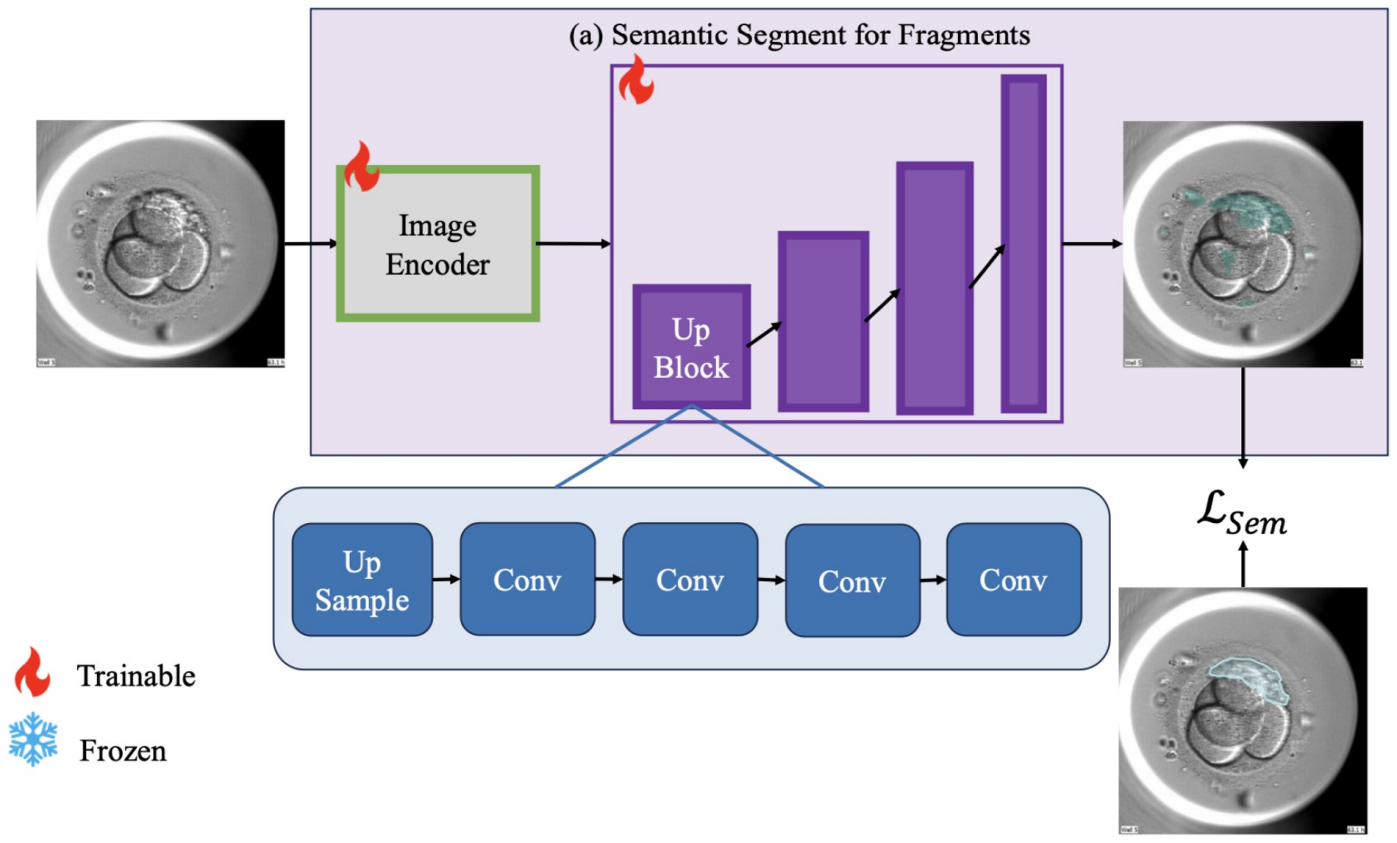
Semantic branch performs semantic segmentation of fragments within the image. The Semantic Decoder is composed of four stacked upsampling modules (UpBlock).

The Semantic Branch, illustrated in Fig. 2, is designed to identify fragments within the input image through semantic segmentation. We construct the Semantic Decoder according to basic semantic segmentation models. The Semantic Decoder comprises a sequence of UpBlocks, which decode the Image Embeddings to the original image resolution. Each UpBlock is implemented with an upsample layer and four cascade convolution layers. This process generates a semantic segmentation mask, in which each pixel is classified as either a fragment or background. During training, the Semantic Branch is optimized using the Sem Loss as follows:

(2) $$L_{\mathrm{Sem}}=L_{\mathrm{Dice}}=1-\frac{2TP}{FP+2TP+FN}$$

where $L_{\mathrm{Dice}}$ is the Dice loss which measures the overlap between predicted and ground truth masks, ensuring accurate segmentation. True Positives (*TP*), False Positives (*FP*), and False Negatives (*FN*) are values from the confusion matrix calculated based on the pixels of predicted and ground truth masks.

### 2.3 Instance segment for blastomeres

**Figure 3.**
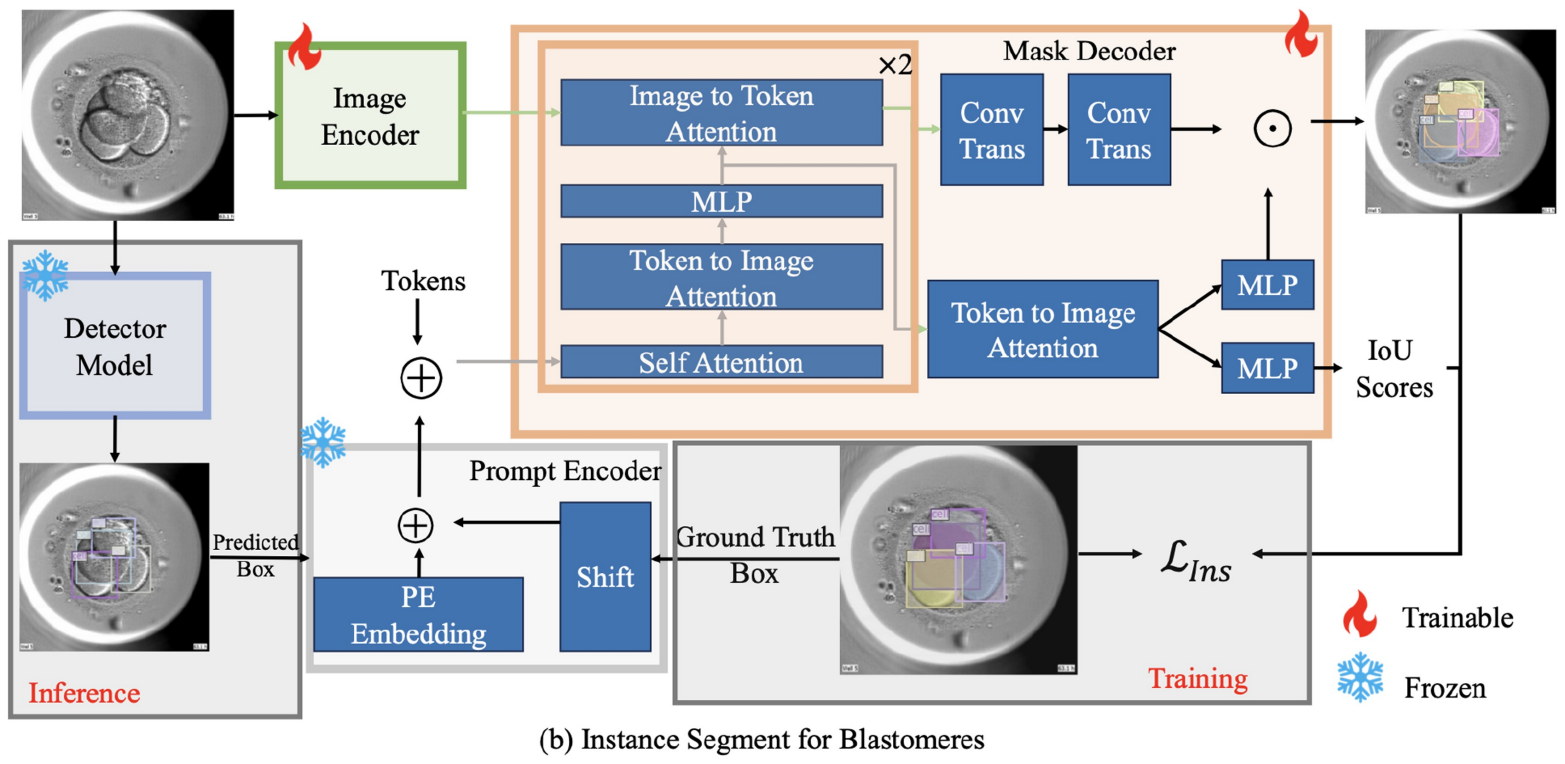
Instance branch focuses on generating precise instance segmentation masks for individual blastomeres. During training, SAM’s Prompt Decoder uses Ground Truth bounding boxes to create Prompt Embeddings, which are combined with Image Embeddings and processed by the Mask Decoder to produce the final instance segmentation masks. During inference, the trained Detector Model generates bounding boxes as prompts.

Inspired by the idea of utilizing a detection model for zero-shot instance segmentation in SAM, we design the Instance Branch, as shown in Fig. 3, to generate precise instance segmentation masks for individual blastomeres using detection-based prompts. During inference, initially, a pre-trained detection model identifies blastomeres and generates bounding boxes. SAM’s Prompt Decoder then processes these bounding boxes to obtain Prompt Embeddings. The Mask Decoder subsequently combines these embeddings with Image Embeddings to create instance segmentation masks for each detected blastomere.

The instance segmentation loss function $L_{\mathrm{Ins}}$ is defined as follow:

(3) $$L_{\mathrm{Ins}}=\sum_{i=1}^{N}\left(L_{\mathrm{Focal}}\left({\tilde{O}}_{z_{i}},O_{z_{i}}\right)+L_{\mathrm{Dice}}\left({\tilde{O}}_{z_{i}},O_{z_{i}}\right)+L_{\mathrm{MSE}}\left({\tilde{\nu }}_{i},\nu_{i}\right)\right)$$

where $L_{\mathrm{Focal}}$ is the Focal loss, used to tackle the issue of class imbalance between background pixels and foreground pixels, ${\tilde{O}}_{z_{i}}$ and $O_{z_{i}}$ represent the predicted and ground truth instance masks for the *i*th instance, respectively. $L_{\mathrm{Dice}}$ is used to improve the quality of the segmentation. $L_{\mathrm{MSE}}$ is the mean squared error loss for the IoU predictions, used to improve the quality of IoU predictions and offer more supervised information, ${\tilde{\nu }}_{i}$ and $\nu_{i}$ represent the predicted IoU value by SAM and the IoU value between the predicted mask and the ground truth mask for the *i*th instance, respectively. The losses are calculated for each predicted instance mask and then summed to get the total loss.

### 2.4 Mask fusion

At the end of the pipeline, the instance segmentation masks for individual blastomeres and the shared semantic segmentation mask for all fragments are combined to produce the final unified segmentation result. Specifically, each blastomere instance has its own unique mask $M_{i}$, where *i* represents the index of each blastomere instance. All fragments are represented by a single, shared mask $M_{f}$. The Mask Fusion process involves combining these masks at the pixel level, which can be formalized as:

(4) $$M_{\text{final}}=\overset{N}{\underset{i=1}{\cup }}M_{i}\cup M_{f}$$

This process ensures that overlapping pixels of each instance in the segmentation are accurately represented in the final segmentation result $M_{\text{final}}$.

## 3 Results

### 3.1 CleavageEmbryo dataset

In this study, we introduce and utilize the CleavageEmbryo dataset, the first to offer pixel-level annotations for human cleavage-stage embryos. This dataset addresses the limitations of existing publicly accessible datasets, which either lack sufficient annotations of cleavage-stage embryos. The CleavageEmbryo dataset includes detailed annotations of cleavage-stage embryos that account for both blastomeres and fragments, making it particularly valuable for training deep-learning models aimed at assessing embryo development.

**Figure 4.**
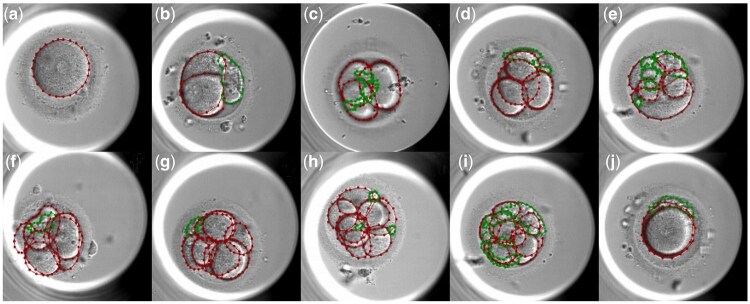
CleavageEmbryo dataset. (a–h) are captured at different stages (1-blastomere to 8-blastomeres, respectively), (i) shows an embryo with >8 blastomeres, and (j) shows a blastomere completely covering another. Red lines represent the manually labeled blastomere boundaries, while green lines represent the manually labeled fragment boundaries.

The CleavageEmbryo dataset consists of high-resolution images of cleavage-stage embryos, captured at various developmental stages, as shown in Fig. 4. Each image in this dataset is a frame extracted from time-lapse videos of embryo development. The images are annotated by four experienced doctors from Renmin Hospital of Wuhan University using the LabelMe (Russell *et al.* 2008), ensuring accuracy and consistency. The CleavageEmbryo dataset has been processed and formatted in the COCO format, which is widely used for object detection and segmentation tasks. This format facilitates the integration and use of our dataset with various deep-learning frameworks and tools.

**Figure 5.**
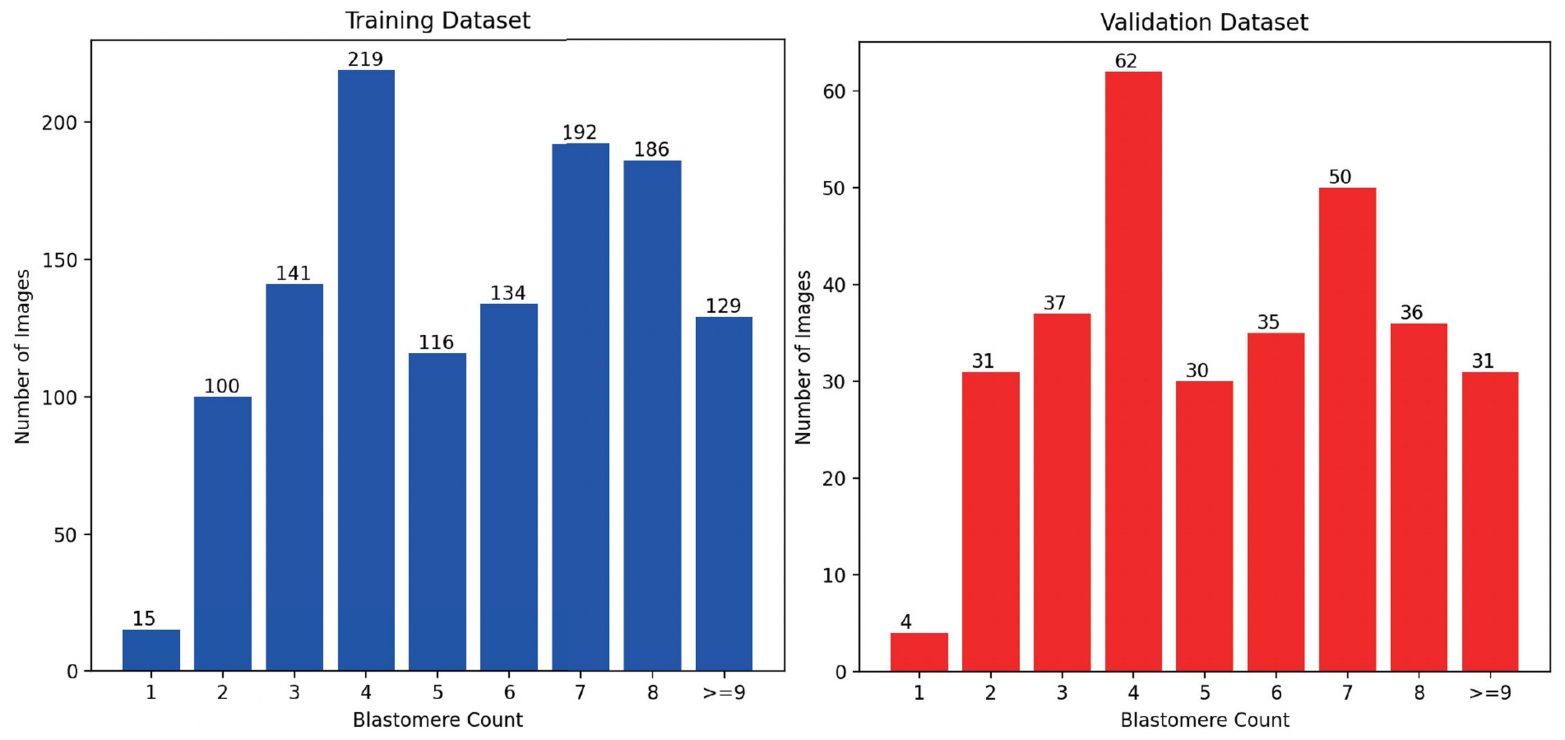
Number of images for each cell cleavage-stage in datasets.

The CleavageEmbryo dataset comprises 1548 images, each with a resolution of 800×800, annotated with pixel-level precision. The dataset is split into a nearly 8:2 ratio for training and evaluation, with 1232 images in the training set and 316 images in the validation set. Figure 5 illustrates the distribution of images across various cell cleavage stages in the training and validation datasets. The annotations cover multiple categories, including:

- **Blastomeres:** Identification and detailed segmentation of individual blastomeres.
- **Fragments:** Segmentation of fragments, which are critical for assessing embryo quality.
- **Background:** Non-embryonic regions.

### 3.2 Experimental settings

#### 3.2.1 Evaluation metrics

To quantitatively evaluate algorithm performance, we use the following metrics:

- **Precision:**
 
  (5) $$\mathrm{Precision}=\frac{TP}{TP+FP}$$
  where True Positives (*TP*) are the number of correctly identified positive instances and False Positives (*FP*) are the number of incorrectly identified positive instances.
- **Recall:**
 
  (6) $$\mathrm{Recall}=\frac{TP}{TP+FN}$$
  where False Negatives (*FN*) are the number of positive instances incorrectly identified as negative.
- **F1-Score:**
 
  (7) $$F1-\mathrm{Score}=2\cdot \frac{\mathrm{Precision}\cdot \mathrm{Recall}}{\mathrm{Precision}+\mathrm{Recall}}$$
- **mAP:**
 
  (8) $$\mathrm{mAP}=\frac{1}{N}\sum_{i=1}^{N}AP_{i}$$
  where *N* is the number of classes and $AP_{i}$ is the average precision for class *i*.
- **Dice:**
 
  (9) $$\mathrm{Dice}=\frac{2TP}{FP+2TP+FN}$$
- **Hausdorff Distance:**
 
  (10) $$HD(P,G)=\max\left\{\underset{p_{i}\in P}{\sup}\underset{g_{i}\in G}{\inf}d(p_{i},g_{i}),\underset{g_{i}\in G}{\sup}\underset{p_{i}\in P}{\inf}d(g_{i},p_{i})\right\}$$
  where $p_{i}$ and $g_{i}$ denote the predicted and ground truth pixels and *P* and *G* represent the sets of $p_{i}$ and $g_{i}$, respectively.

For detection, the TP, FP, and other related metrics are computed based on the comparison of bounding boxes with a 50% IoU threshold, i.e. TP is counted when the IoU between the predicted and ground truth bounding boxes exceeds 50%. For segmentation, the metrics are computed at the pixel level, i.e. whether the pixel is correctly or incorrectly classified.

#### 3.2.2 Training configuration

During training, since there is little or almost no similar image information involved in the training process of SAM, we fine-tune the Image Encoder and Mask Decoder while freezing the Prompt Decoder, as it only utilizes bounding box coordinates. Specifically, we use real bounding boxes of blastomeres to guide the fine-tuning. At the same time, we initially trained the Semantic Decoder together, using the ground truth masks of fragments.

Considering the input size of the SAM is 1024×1024 while the resolutions of images in our dataset are 800×800, we applied padding to both images and labels during training to resize them to the required size. During inference, we pad the image to the required size and then resize the resulting masks back to the original image size. Other settings of hyperparameters are described in Supplementary Information.

The Detector Models which are used to generate accurate bounding boxes as prompts required by blastomeres segment, are trained separately. We train and test several mainstream and advanced object detection algorithms on the CleavageEmbryo dataset and then choose the best one of them, including FCOS (Tian *et al.* 2019), DETR (Carion *et al.* 2020), YOLOX (Ge *et al.* 2021), Yolov8, Dino (Zhang *et al.* 2022), and Co-DETR (Zong *et al.* 2023). We evaluate their performance in detecting blastomeres and fragments using Precision, Recall, F1-Score, and mAP metrics.

We select the best-performing object detection algorithm (Yolov8) and integrate it into our pipeline for blastomere segmentation. Additionally, we train and test state-of-the-art instance segmentation algorithms on our dataset, including Cascade Mask R-CNN (Cai and Vasconcelos 2021), Mask Scoring RCNN (Huang *et al.* 2019), YOLACT (Bolya *et al.* 2019), SOLOv2 (Wang *et al.* 2020), Mask2Former (Cheng *et al.* 2021), RTMDet (Lyu *et al.* 2022), DoNet (Jiang *et al.* 2023), and GAInS (Liu *et al.* 2024). We evaluate them using mAP, Precision, Recall, and F1-Score metrics. Yolov8 are trained on our dataset initially and other models mentioned above are initialized with pre-trained checkpoints which are trained on COCO datasets.

To further demonstrate the effectiveness of SAM-based semantic segmentation for fragments, we train and test several semantic segmentation models on fragment segmentation, including UNet, UNet++ (Zhou *et al.* 2020), DeepLabV3 (Chen *et al.* 2017), SegFormer (Xie *et al.* 2021), and TransUnet (Chen *et al.* 2021). We evaluate these models using Dice and Hausdorff distance metrics. These models are trained initially on our datasets. More implementation details can be found in Supplementary Information.

### 3.3 Comparison with SOTA methods

**Figure 6.**
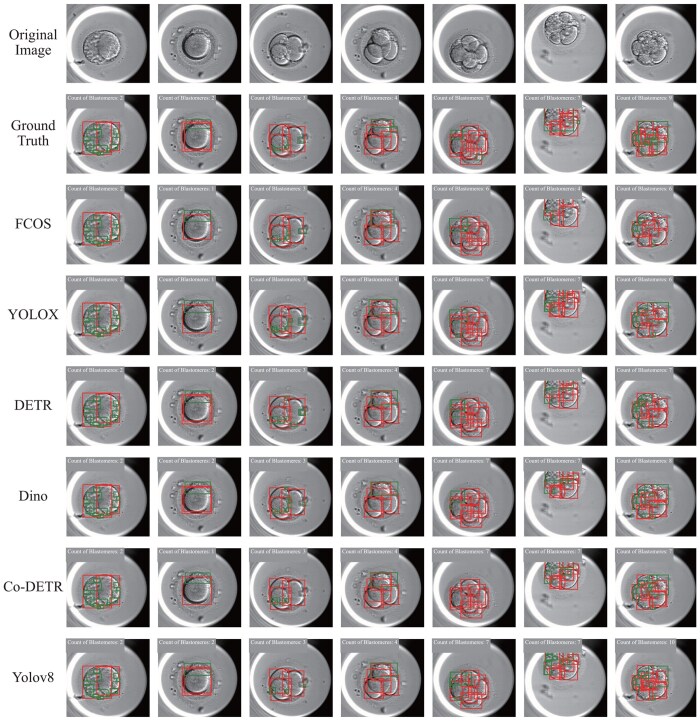
Visualization of object detection results for each model on the CleavageEmbryo Dataset. The red boxes indicate blastomeres and the green boxes represent fragments.

In Fig. 6, we visualize the object detection results. Table 1 presents the quantitative results. Yolov8 outperforms other models in both blastomere and fragment detection, achieving the highest Precision (0.648) and mAP (0.874) on blastomeres detection, and similarly higher metrics for fragments. Therefore, we select Yolov8 as the detector to generate bounding boxes for blastomeres in our pipeline. Nevertheless, it is important to note that even the best object detection model shows poor performance on fragment detection, which will greatly affect segmentation results generated by SAM’s Mask Decoder due to the lack of precise prompts. Consequently, we adapt semantic segment and create the Semantic Decoder to generate accurate fragment segmentation.

**Table 1.** Quantitative results of the object detection models on the CleavageEmbryo dataset.

	Blastomere				Fragment			
Methods	Precision↑	Recall↑	F1-score↑	mAP↑	Precision↑	Recall↑	F1-score↑	mAP↑
FCOS	0.524	0.534	0.625	0.689	0.299	0.318	0.415	0.377
YOLOX	0.602	0.610	0.674	0.806	0.292	0.310	0.386	0.375
DETR	0.596	0.609	0.662	0.794	0.187	0.219	0.287	0.196
Dino	0.645	**0.655**	0.708	0.867	0.313	**0.353**	0.423	0.375
Co-DETR	0.645	0.654	**0.709**	0.871	0.330	0.353	**0.440**	0.420
Yolov8	**0.648**	**0.655**	0.708	**0.874**	**0.333** ^a^	0.352	0.425	**0.436**

a The bold value means the beast one.

**Figure 7.**
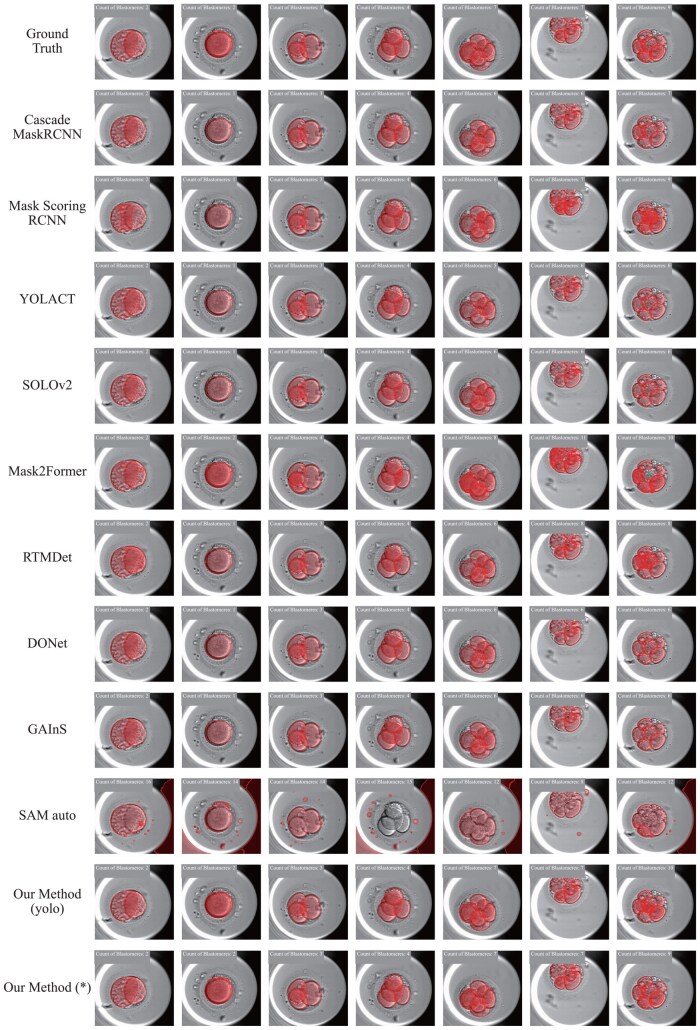
Visualization of blastomeres segmentation results. (SAM auto means we generate prompt automatically without the help of Detector Model, and * means we use ground truth bounding box as prompt).

In Fig. 7, we present the visualization of blastomere segmentation results. Table 2 presents the quantitative results of various instance segmentation models, illustrating the superior performance of our proposed pipeline for blastomere segmentation. Specifically, with Yolov8, our method achieves excellent Precision (0.647), Recall (0.653), F1-Score (0.706), and mAP (0.874). Moreover, with the ground truth bounding box as prompt, our method achieves the highest Precision (0.694), Recall (0.702), F1-Score (0.748), and mAP (0.942) among all evaluated models, highlighting that we can still achieve better segmentation results by improving detection performance.

**Table 2.** Quantitative results of blastomere segmentation (auto means we generate prompt automatically without the help of Detector Model, and * means we use ground truth bounding box as prompt).

	Blastomere			
Methods	Precision↑	Recall↑	F1-score↑	mAP↑
Cascade MaskRCNN	0.560	0.566	0.654	0.745
Mask Scoring RCNN	0.508	0.515	0.582	0.672
YOLACT	0.494	0.507	0.602	0.643
SOLOv2	0.575	0.581	0.652	0.764
Mask2Former	0.534	0.540	0.600	0.708
RTMDet	0.568	0.575	0.646	0.756
DONet	0.564	0.570	0.658	0.750
GAInS	0.545	0.553	0.639	0.723
SAM auto	0.002	0.039	0.034	0.002
Our method (Yolov8)	**0.647**	**0.653**	**0.706**	**0.874**
Our method (*)	**0.694** ^a^	**0.702**	**0.748**	**0.942**

a The bold value means the beast one.

**Figure 8.**
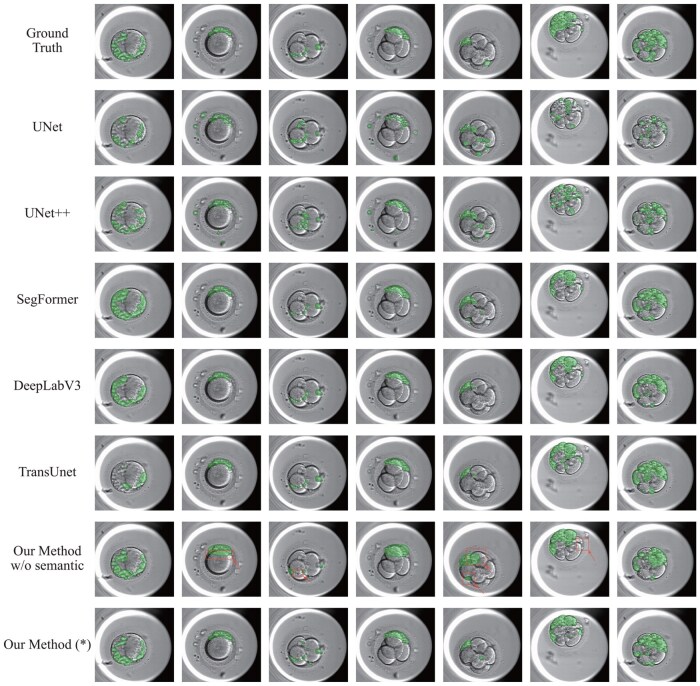
Visualization of fragments segmentation results. In the visualization results of Our Method w/o semantic, red arrows and boxes indicate mis or incorrectly detected fragments.

In Fig. 8, we present a visualization of the results of fragment segmentation. Table 3 provides the quantitative results of the performance of various semantic segmentation models. Both the visualization and quantitative results indicate that segmenting fragments is a challenging task, and there is considerable scope for improvement.

**Table 3.** Quantitative results of the fragment segmentation.

	Fragment	
Models	Dice↑	Hausdorff↓
UNet	0.290	193.375
UNet++	0.315	203.040
DeepLabV3	0.677	77.451
SegFormer	0.672	**73.731**
TransUnet	0.624	102.929
Our method w/o semantic	0.667	79.076
Our method	**0.695** ^a^	78.353

a The bold value means the beast one.

Overall, the visualizations validate quantitative results, demonstrating our method delivers more accurate blastomere and fragment segmentation—particularly in complex cases involving overlapping cells and small fragments.

### 3.4 Ablation study

To validate the effectiveness of the Detector Model, we conduct an ablation study in which we adapt the strategy of SAM’s automatically generating prompts to generate masks of instances without the Detector Model. As shown in Table 2 and Fig. 7, SAM performs poorly in this case, demonstrating it cannot identify the category of the instance mask without the Detector Model.

To validate the effectiveness of the Semantic Decoder, we apply the same approach used for instance segmentation on blastomeres to generate the fragment segmentations. Without the Semantic Branch, the Detection Model would overlook or incorrectly detect fragments, as shown in Fig. 8, leading to poor fragment segmentation. Quantitative metrics in Table 3 also indicate that adding the Semantic Branch leads to higher Dice (0.695) and lower Hausdorff distances (78.353), confirming the visual observations.

In summary, the presence of the Detector Model is crucial for accurate instance detection and classification, while the incorporation of the Semantic Branch into our pipeline enhances the quality of the fragment segmentation. These studies highlight the effectiveness of our approach in tackling the challenges of embryo segmentation.

### 3.5 Test on time-lapse videos

**Figure 9.**
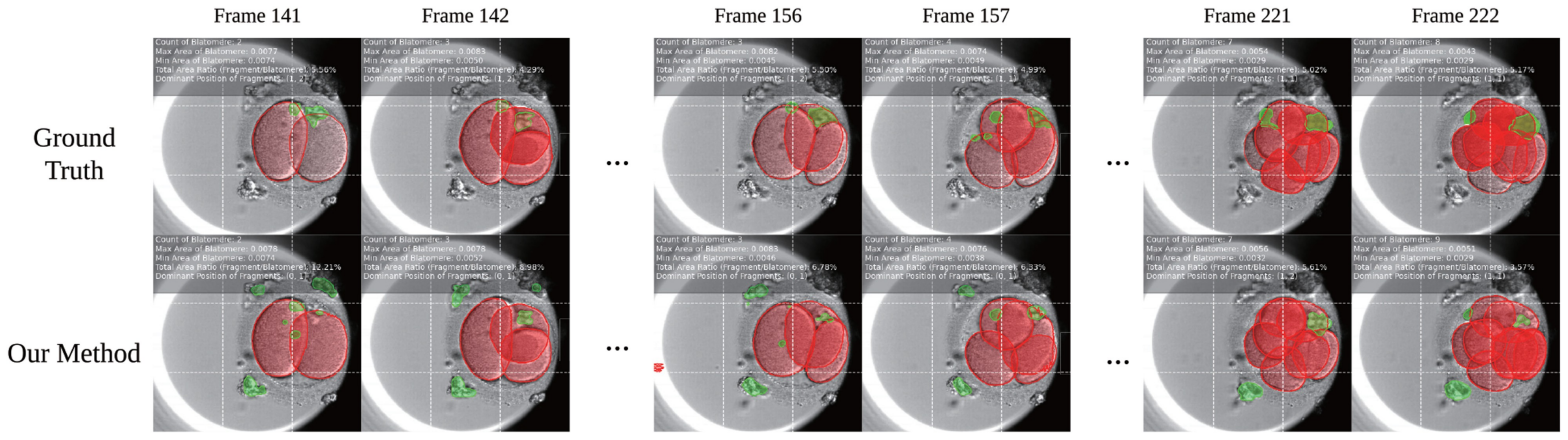
Visualization of the segmentation results of keyframes in time-lapse videos. Red masks indicate blastomeres and green masks indicate fragments. The text on the images shows the number of blastomeres, the maximum and minimum blastomere area relative to the image’s resolution, the ratio of fragment area to blastomere area, and the dominant position of fragments.

To effectively capture the morphokinetic information, such as the starting times for cell cleavage stages and cell movement trajectories, which are crucial for assessing embryo viability and implantation potential, we extend our research to the time-lapse videos. By segmenting the entire videos and applying post-processing techniques, we can extract keyframes and obtain the segmentation results, as illustrated in the Fig. 9. This approach can aid doctors in observing the embryo cleavage process and the distribution of fragments, offering detailed insights into the dynamics of embryonic development.

We can extract key morphological information such as the maximum of blastomeres and the dominant position of fragments from segmentation results. These characteristics are crucial for evaluating the developmental potential of embryos.

In summary, the visualization results from our experiments highlight the effectiveness of our approach in time-lapse video analysis. This application extension of our research enables more comprehensive monitoring and evaluation of embryonic development, thereby supporting clinical decision-making.

## 4 Conclusion

In conclusion, our study introduces the new SAM-based Dual Branch Segmentation Pipeline for the automated segmentation of cleavage-stage embryos, addressing a critical need to improve the efficiency and objectivity of embryo assessment in IVF. By leveraging SAM’s robust segmentation capabilities, our method combines instance and semantic segmentation to accurately segment both blastomeres and fragments within embryos. This approach represents a significant advancement over the existing methods, as it achieves superior performance in both quantitative metrics and visual quality.

Importantly, the development of the CleavageEmbryo dataset, with detailed pixel-level annotations, represents a pioneering effort in the field. This dataset will enable future research and benchmarking in automated embryo assessment. To further advance the field of deep learning-based embryo assessment, expanding the CleavageEmbryo dataset is crucial. We plan to include more developmental stages, covering the entire process from the early cleavage-stage to the blastocyst stage, and to record the final clinical outcomes of each embryo, which can be used to train and evaluate more robust and generalizable AI models. In addition, though the Semantic Decoder in our pipeline meets the basic requirements for segmentation, it does not fully exploit the potential. Future work will also focus on exploring more advanced strategies to apply SAM to semantic segmentation.

## Author contributions

Chensheng Z, Jianhui Z, Yi Z: Writing; Xintong S, Xinyue Y, Jiayi S, Yi Z: Data Curation; Chensheng Z, Jianhui Z: Methodology.

## Supplementary Material

btae617_Supplementary_Data

## Data Availability

The data underlying this article are available in the article and in its online supplementary material.
