# Expression of Concern: Cleavage-Stage Embryo Segmentation Using SAM-Based Dual Branch Pipeline: Development and Evaluation with the CleavageEmbryo Dataset

**DOI:** 10.1093/bioinformatics/btaf001

**Published:** 2025-01-11

This is an expression of concern regarding: Chensheng Zhang, Xintong Shi, Xinyue Yin, Jiayi Sun, Jianhui Zhao, Yi Zhang, Cleavage-Stage Embryo Segmentation Using SAM-Based Dual Branch Pipeline: Development and Evaluation with the CleavageEmbryo Dataset, *Bioinformatics*, 2024, btae617, https://doi.org/10.1093/bioinformatics/btae617

Shortly after publication of the accepted manuscript, the authors notified the journal that subsequent revision of their data changed the results presented and reported. While the authors update the text and figures accordingly, and the journal reviews the proposed changes, the editors advise readers to examine the details of this study with particular care.

