# Publisher’s Note: ‘Expression of Concern: Cleavage-Stage Embryo Segmentation Using SAM-Based Dual Branch Pipeline: Development and Evaluation with the CleavageEmbryo Dataset’

**DOI:** 10.1093/bioinformatics/btag625

**Published:** 2026-08-27

This is a Publisher’s Note regarding ‘Expression of Concern: Cleavage-Stage Embryo Segmentation Using SAM-Based Dual Branch Pipeline: Development and Evaluation with the CleavageEmbryo Dataset’, *Bioinformatics*, Volume 41, Issue 1, January 2025, https://doi.org/10.1093/bioinformatics/btaf001

This Expression of Concern applied to the Accepted Manuscript version of the paper. The necessary changes were incorporated into the corrected and typeset Version of Record at https://doi.org/10.1093/bioinformatics/btae617.

