# Supplementary material for: Cleavage-stage embryo segmentation using SAM-based dual branch pipeline: development and evaluation with the CleavageEmbryo dataset
Source: Bioinformatics. 2024 Oct 18;41(4):btae617. doi: 10.1093/bioinformatics/btae617 (PMC11955249; doi:10.1093/bioinformatics/btae617)
Supplement: btae617_Supplementary_Data [file btae617_supplementary_data.docx]

Supplementary information:

**CleavageEmbryo Dataset:**

1. download URL: <https://www.kaggle.com/datasets/austin012/cleavageembryo-dataset/data>
2. Information:
   1. Source: First School of Clinical Medicine, Wuhan University.
   2. Annotation: The images are annotated by three experienced doctors from Renmin Hospital of Wuhan University using the LabelMe.
   3. Categories:

**Blastomeres:** Detailed segmentation of individual blastomeres.

**Fragments:** Identification and segmentation of fragments, which are critical for assessing embryo quality.

**Background:** Non-embryonic regions to assist in accurate segmentation.

**Implementation Details:**

1. code: <https://github.com/12austincc/Cleavage-StageEmbryoSegmentation>
2. config:
   1. optimizer: Adam
   2. Learning rate:

for image encoder and mask decoder: 3e-5

for semantic decoder:3e-5

- 1. batch_size:2

environment: NVIDIA GeForce RTX 3090 X1

3、 Training configuration for other models:

Object detection:

fcos: backbone: resnet101; optimizer: SGD; lr: 0.01, weight_decay: 0.0001

detr: backbone: resnet101; optimizer: AdamW; lr: 0.01, weight_decay: 0.0001

yolox: backbone: resnet101; optimizer: SGD; lr: 0.01, weight_decay: 0.0005

dino: backbone: resnet101; optimizer: AdamW; lr: 0.01, weight_decay: 0.0001

co-detr: backbone: resnet101; optimizer: AdamW; lr: 0.0001, weight_decay: 0.0001

Blastomere segmentation:

cascade maskrcnn: backbone:resnet101; optimizer: SGD; lr: 0.01, weight_decay: 0.0001

mask scoring rcnn: backbone:resnet101; optimizer: SGD; lr: 0.02, weight_decay: 0.0001

yolact: backbone:resnet101; optimizer: SGD; lr: 0.001, weight_decay: 0.0005

solov2: backbone:resnet101; optimizer: SGD; lr: 0.001, weight_decay: 0.0001

mask2former: backbone:resnet101; optimizer: AdamW; lr: 0.0001, weight_decay: 0.05

rtmdet: backbone:resnet101; optimizer: AdamW; lr: 0.004, weight_decay: 0.05

donet: backbone:resnet101; optimizer: AdamW; lr: 0.004, weight_decay: 0.05

Semantic segmentation:

unet: backbone: resnet101; optimizer: SGD; lr: 0.01, weight_decay: 0.001

unet++: backbone: resnet101; optimizer: SGD; lr: 0.01, weight_decay: 0.001

deeplabv3: backbone: resnet101; optimizer: SGD; lr: 0.001, weight_decay: 0.001

segformer: backbone: mit; optimizer: AdamW; lr: 0.00006, weight_decay: 0.01

4、model details:

5、Data transform:

while training: pad to 1024 * 1024 for images and masks

while inferencing:

pad to 1024*1024 for images; remove padding and upscale to 800*800 for generated masks;

while evaluating:

for fragments: remove image id without instances of fragments:

[12,20,21,22,30,34,37,38,56,67,71,73,83,84,96,104,110,119,135,141,143,148,155,

158,160,169,174,176,179,197,203,208,213,227,228,238,247,258,265,267,281,292,297,323,330, 351,353,358,363,367,370,392,394]

remove image id with fault annotation: [109,65,365,305,243,416,310,126]
